# Unique Role of Vimentin Networks in Compression Stiffening of Cells and Protection of Nuclei from Compressive Stress

**DOI:** 10.1021/acs.nanolett.2c00736

**Published:** 2022-06-09

**Authors:** Katarzyna Pogoda, Fitzroy Byfield, Piotr Deptuła, Mateusz Cieśluk, Łukasz Suprewicz, Karol Skłodowski, Jordan L. Shivers, Anne van Oosten, Katrina Cruz, Ekaterina Tarasovetc, Ekaterina L. Grishchuk, Fred C. Mackintosh, Robert Bucki, Alison E. Patteson, Paul A. Janmey

**Affiliations:** † Institute of Nuclear Physics Polish Academy of Sciences, Krakow PL-31-342, Poland; ‡ Department of Physiology, and Institute for Medicine and Engineering, University of Pennsylvania, Philadelphia, Pennsylvania 19104-6383, United States; § Department of Medical Microbiology and Nanobiomedical Engineering, Medical University of Bialystok, PL-15222 Bialystok, Poland; ∥ Department of Chemical and Biomolecular Engineering, Rice University, Houston, Texas 77005, United States; ⊥ Center for Theoretical Biological Physics, Rice University, Houston, Texas 77030, United States; # Department of Physiology, University of Pennsylvania, Philadelphia, Pennsylvania 19104-6383, United States; ∇ Departments of Chemistry and Physics and Astronomy, Rice University, Houston, Texas 77005, United States; ○ Department of Physics and BioInspired Institute, Syracuse University, Syracuse, New York 13210, United States

**Keywords:** intermediate filaments, vimentin, compression-stiffening, AFM, compressive stress

## Abstract

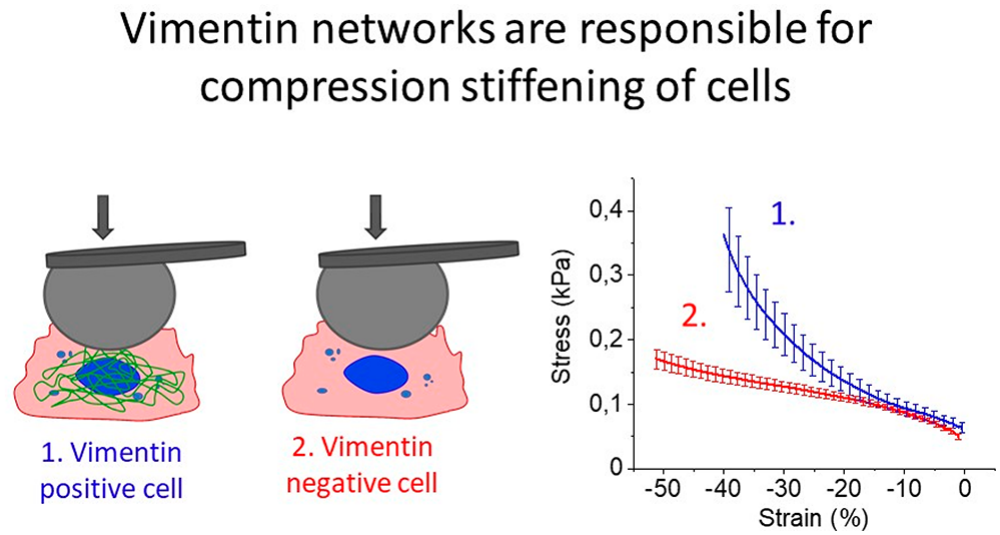

In this work, we investigate whether stiffening in compression is a feature of single cells and whether the intracellular polymer networks that comprise the cytoskeleton (all of which stiffen with increasing shear strain) stiffen or soften when subjected to compressive strains. We find that individual cells, such as fibroblasts, stiffen at physiologically relevant compressive strains, but genetic ablation of vimentin diminishes this effect. Further, we show that unlike networks of purified F-actin or microtubules, which soften in compression, vimentin intermediate filament networks stiffen in both compression and extension, and we present a theoretical model to explain this response based on the flexibility of vimentin filaments and their surface charge, which resists volume changes of the network under compression. These results provide a new framework by which to understand the mechanical responses of cells and point to a central role of intermediate filaments in response to compression.

Cells and tissues are subjected to different types of external mechanical stresses, including compressive loads, pressure gradients, or shear. Although the fibrous cytoskeleton provides the mechanical strength of an individual cell,^1^ previous studies have shown that tissues’ resistance to mechanical stress primarily depends on the extracellular matrix (ECM)^2^ with the cells included within it limiting the relaxation modes available to the ECM.^3,4^ Extracellular matrices formed by collagen or fibrin alone can resist shear and elongation strains and become stiffer with increasing deformation, but in compression they become softer because stiff filaments buckle in compression.^5−8^ Inclusion of particles larger than the matrix mesh size^3,4^ or rigid cross-links within a highly connected network^9^ suppress buckling and promote compression stiffening.^10^ Whether similar effects occur in single cells is unknown. Here, we compare the responses of purified cross-linked networks formed by F-actin, microtubules, and vimentin intermediate filaments and show that actin and MT networks soften in compression, but vimentin networks stiffen. The unique effects of vimentin networks are consistent with a theoretical model, and their contribution to whole cell mechanics is evident in studies of a single mEF cell compression using AFM. Mechanical testing of nuclei isolated from vimentin expressing and vimentin null cells show that this effect is not the result of nucleus compression but suggests that the vimentin networks that surround the nucleus enable compression-stiffening of cells and protect the nucleus from mechanical damage.

## Responses of Cytoskeletal Networks to Uniaxial Compression

**Figure 1:**
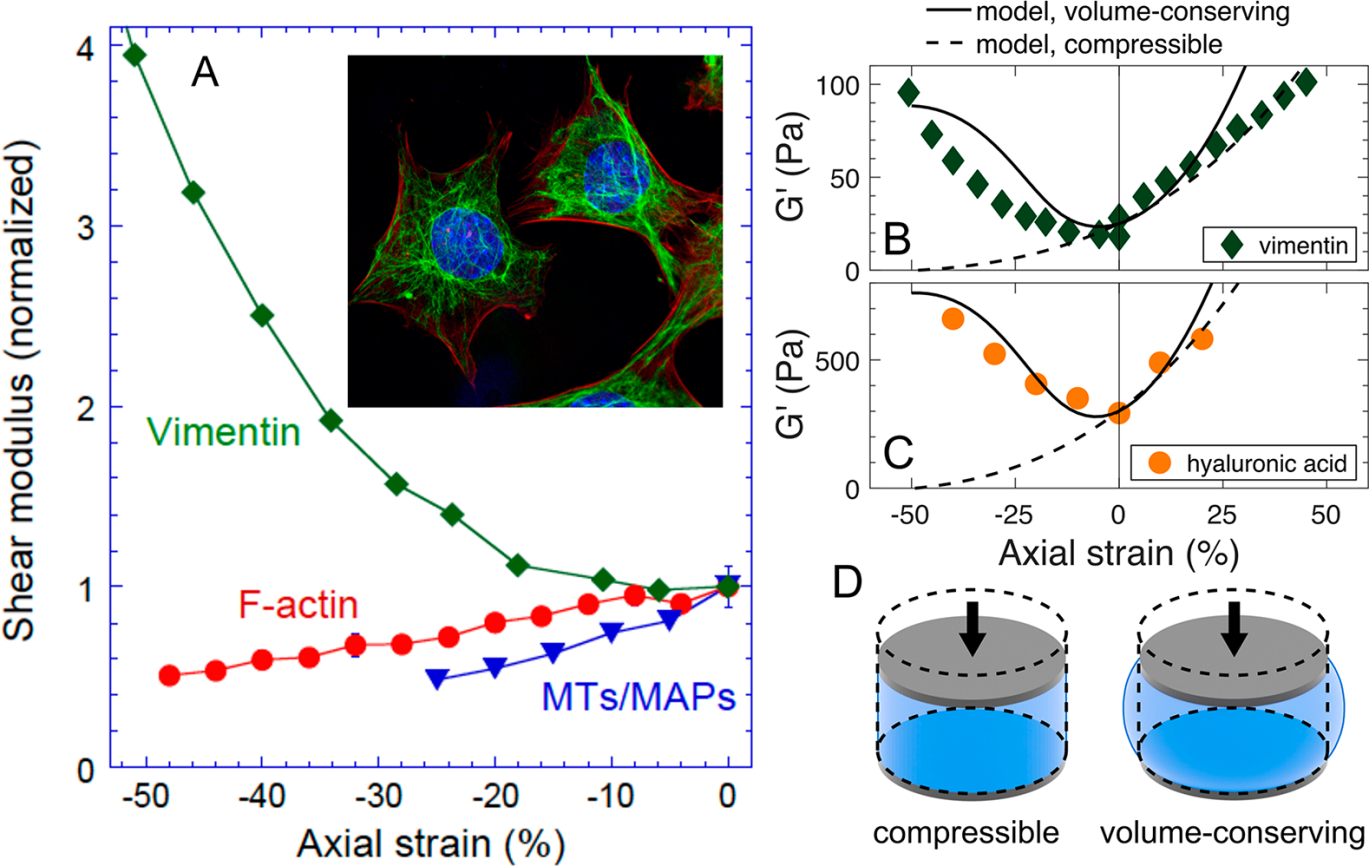
Cytoskeletal polymer networks response to uniaxial compression. (A) Compression stiffening of vimentin and compression-softening of F-actin and microtubules network with increasing axial strain as measured using strain rheometer. (B,C) Both hyaluronic acid networks and vimentin networks stiffen in extension and compression. This behavior is recapitulated by the theoretical model when incompressibility is assumed. (D) Schematic illustration of compressible and volume-conserving samples.

When cross-linked networks of actin filaments or microtubules are subject to increasing compression, the shear modulus measured at low strain decreases as the axial strain magnitude becomes larger (more negative) (Figure 1A). The softening of these two cytoskeletal networks is similar to the responses of collagen and fibrin networks,^7^ as well as those of synthetic semiflexible polymer networks,^11^ and is explained by the buckling of stiff filaments within compressed networks.^6^ In contrast, networks of vimentin intermediate filaments (VIFs) become stiffer as they are compressed, as shown by the increase in shear modulus in response to axial strain. The abundance of VIFs within mesenchymal cells such as the fibroblast shown in the inset of Figure 1A suggests that unlike actin filaments, which dominate the stiffness of the cell cortex, intermediate filaments might dominate the mechanical response of cells undergoing global compression. The response of VIF networks to uniaxial strains is shown in more detail in Figure 1B. Increased shear modulus occurs with both compression and elongation. This stiffening in both compression and extension is not unique to VIF networks but is also seen in cross-linked networks of hyaluronic acid (Figure 1C),^12^ a highly charged semiflexible polysaccharide that is often interspersed in the ECM with collagen and is the dominant ECM element in tissues such as the brain and the vitreous body.^13^

The compression softening of semiflexible filament networks coincides with decreased volume, as solvent is expelled from the compressed network, and requires that the persistence length of the filament be larger than the network mesh size.^6,7^ VIFs differ from F-actin and microtubules in that they have a significantly larger negative surface charge,^14^ and they are at least an order of magnitude more flexible.^15^ Consequently, networks of VIFs might resist changes in volume because of the osmotic effect of their mobile counterions, and because their persistence length is no longer larger than the network mesh size. When the increased flexibility of the VIFs and the incompressibility of the network are considered in the theoretical model (Figure 1D), the large increases in shear modulus in both compression and extension are predicted to be a generic feature of such networks, including vimentin and hyaluronic acid.

## Compression Stiffening of Cells

**Figure 2:**
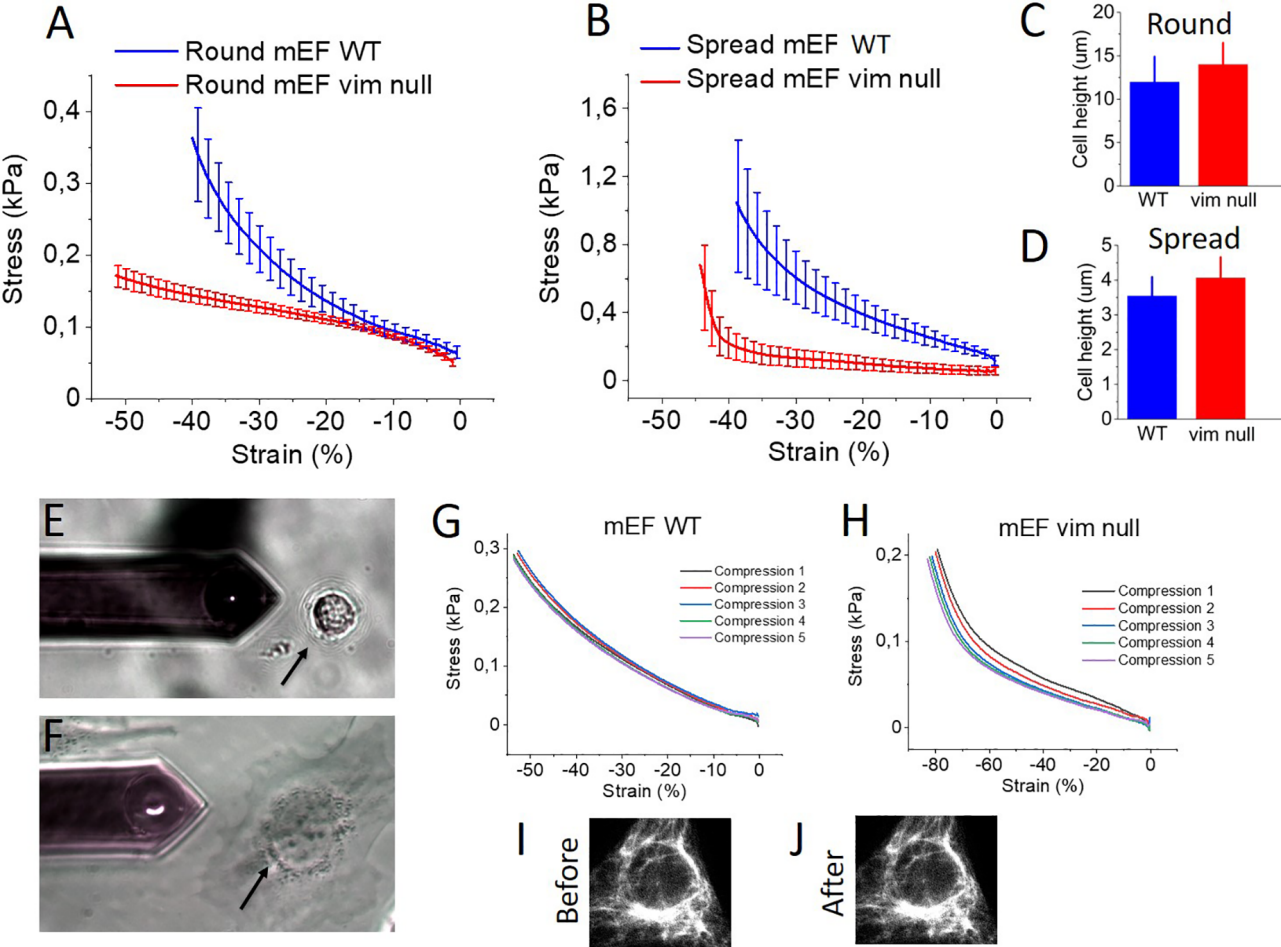
Mechanical response of single wild type and vimentin null mEFs under compressive force. (A,B) Axial stress versus axial strain curves for round and spread wild type mEF and vimentin null mEFs. (C,D) Cell height of round and spread cells prior to AFM uniaxial compression. (E,F) Optical images of the round and spread cells that were used to position the AFM tip before uniaxial compression, taken using 10× objective. (G) The effect of five successive compressions of the mEF WT cells on the stress–strain curve characteristics. (H) The effect of five successive compressions of the mEF vimentin null cells on the stress–strain curve characteristics. (I) Normalized shear stress at 50% strain as a function of consecutive compression cycle for mEF WT and vim null. (J.K) Fluorescence images of the live NIH 3t3 fibroblast expressing GFP vimentin before and after application of the compressive force using AFM.

To determine if single cells change stiffness in compression, wild type and vimentin null fibroblasts were compressed with a 25 μm diameter bead. Experiments were performed using both rounded cells (Figure 2E) and cells that flatten out on a substrate for 24 h (Figure 2F). Rounded cells can be treated as the objects with more uniform internal composition, whereas cell spreading requires rearrangement of the cytoskeletal fibrous networks.

Figure 2A shows that when round cells are compressed by 50 nN force, axial stress rises with axial strain magnitude approximately linearly up to 10% compression for normal mouse embryo fibroblasts (mEFs) and then increases nonlinearly, implying compression stiffening. In contrast, round mEFs from vimentin null mice did not exhibit compression stiffening and have a linear elastic response up to 50% compression. Spread normal mEFs were compressed over the nuclear region where vimentin accumulates, and the compression-stiffening response was similar to that of round cells (Figure 2B). Spread vimentin null mEFs maintained a linear increase of axial stress with axial strain up to 40% compression, after which rapid stiffening was observed, probably due to mechanical response of the cell nucleus. Figure 2C,D shows that there is no difference in the cell height of normal and vimentin null mEFs, and therefore the difference in stiffening behavior cannot be attributed to a possible height-dependent influence of the rigid substrate under the cells. These large compressive forces do not damage WT mEFs, and compression cycles can be repeated five times without weakening of the cell (Figure 2G,H). In contrast, vim null mEFs soften with each compression cycle, consistent with earlier reports that vimentin is required for cell elastic recovery (Figure 2I).^16^

To test how uniaxial compressive force is transferred to the cagelike vimentin network surrounding the cell nucleus, we used NIH 3t3 fibroblasts expressing GFP-vimentin. Time-lapse images of the fluorescently labeled vimentin network inside the cell was recorded simultaneously with AFM compression, allowing for correlation of the image of real-time vimentin cage deformation (see Supplementary Movie 1) with the amount of the compressive force applied. Figure 2J,K was taken from the GFP-vimentin expressing fibroblast just before (Figure 2J) and after (Figure 2K) application of 50 nN compressive force, showing that no visible vimentin cage change. This result also suggests that the rate of deformation is faster than any possible modifications caused by cell signaling, which has been shown to post-translationally alter VIF network mechanics,^17−19^ and the observed difference in compressibility between WT mEFs and vim null mEFs cannot be attributed to such alterations.

## Compression Stiffening of Cells Is Not Solely the Result of Nuclear Deformation

The differences between compression-stiffening of WT and vimentin null cells cannot unambiguously be attributed to the VIF network, because the nucleus might also become deformed at large compressions. To determine if differences in nuclear mechanics could affect the difference in the compression of cells without vimentin, nuclei were isolated from both wild type and vimentin null cells by gentle centrifugation of nuclei from cells attached to a surface orthogonal to the direction of centrifugal force.

**Figure 3:**
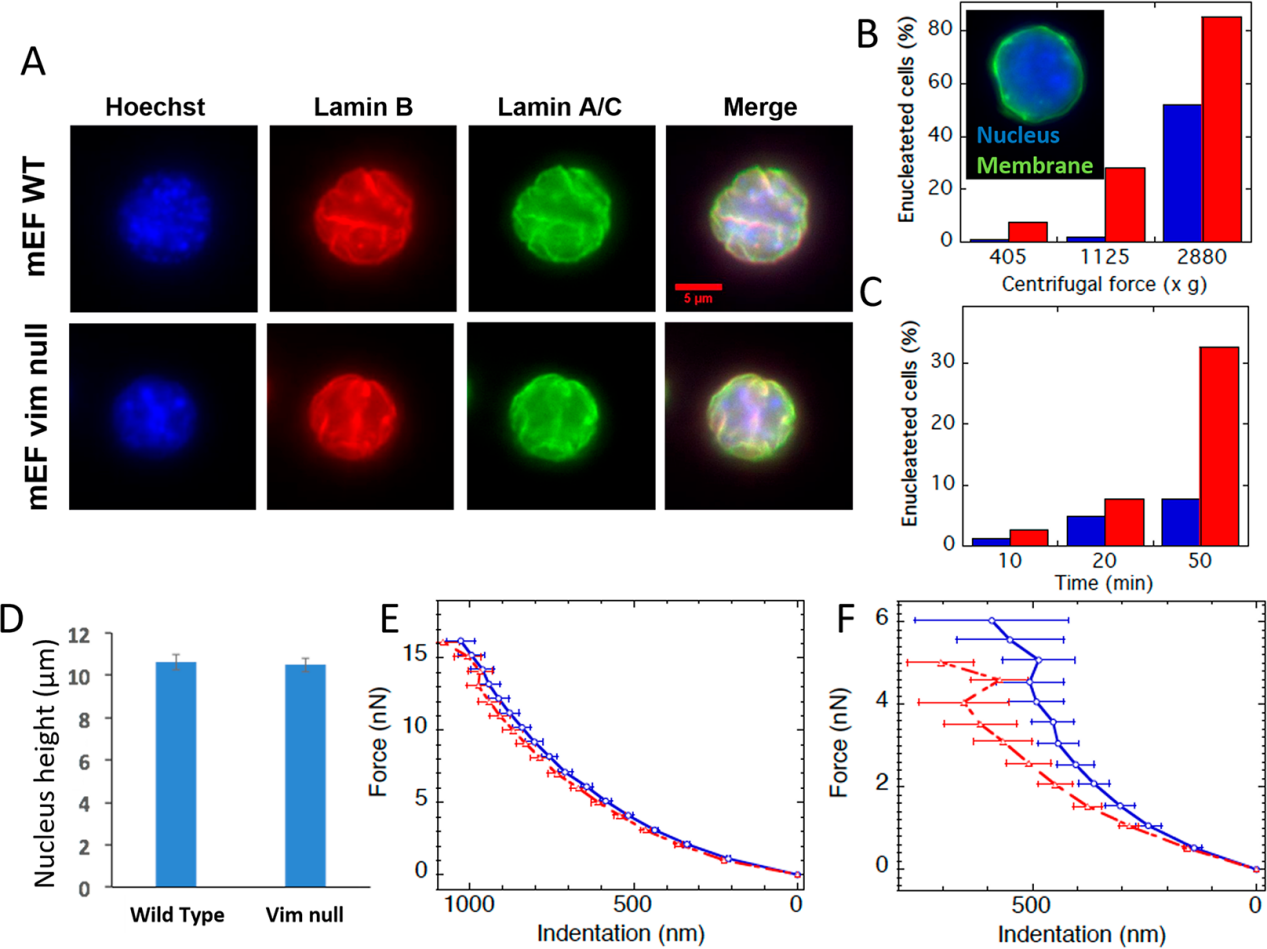
Mechanical response of a single nucleus isolated from the wild type and vimentin null mEFs. (A) Fluorescence staining of the nucleus isolated from wild type and vimentin null mEFs (scale bar 5 μm). (B) Percent of the enucleated cells as a function of centrifugal force. (C) Percent of enucleated cells as a function of centrifugation time. (D) Nucleus height of the wild type mEF and vim null mEFs as determined using AFM. (E) Force versus indentation curve for the nucleus isolated from wild type mEFs (blue) and vim null mEF (red). (F) Force versus indentation curve for the nucleus-free cytoplasts from wild type mEFs (blue) and vim null mEFs (red).

As shown in Figure 3A, nuclei from WT and vim null mEF cells have similar morphology and nuclear lamin arrangement, which is consistent with previous reports of similar expression of lamins A, B1/2, and C for both cell types.^20^Figure 3B,C shows that removal of nuclei from vimentin null cells occurs more readily than from wild type cells. The isolated nuclei maintain an intact plasma membrane and a thin layer of cytoplasm around the nuclear membrane but no detectable cytoskeleton (inset to Figure 3B). Nuclei isolated from wild type and vimentin null cells have statistically the same height (Figure 3D) and their responses to compressive force are indistinguishable (Figure 3E). In contrast, the nucleus-free cytoplasts from wild type cells are stiffer than cytoplasts from vimentin null cells (Figure 3F). These results suggest that differences in whole cell susceptibility to compression are not the result of differences in the nuclei but rather differences due to the presence or absence of vimentin intermediate filament network.

## Protection of Nuclei by Vimentin in Cells under Compression

To determine a possible biological significance of the unique compression stiffening behavior of VIF networks, monolayers of cells were subjected to varying amounts of compressive stress, and the morphology and nuclear integrity of the cells were quantified (Supplementary Figure 1).

**Figure 4:**
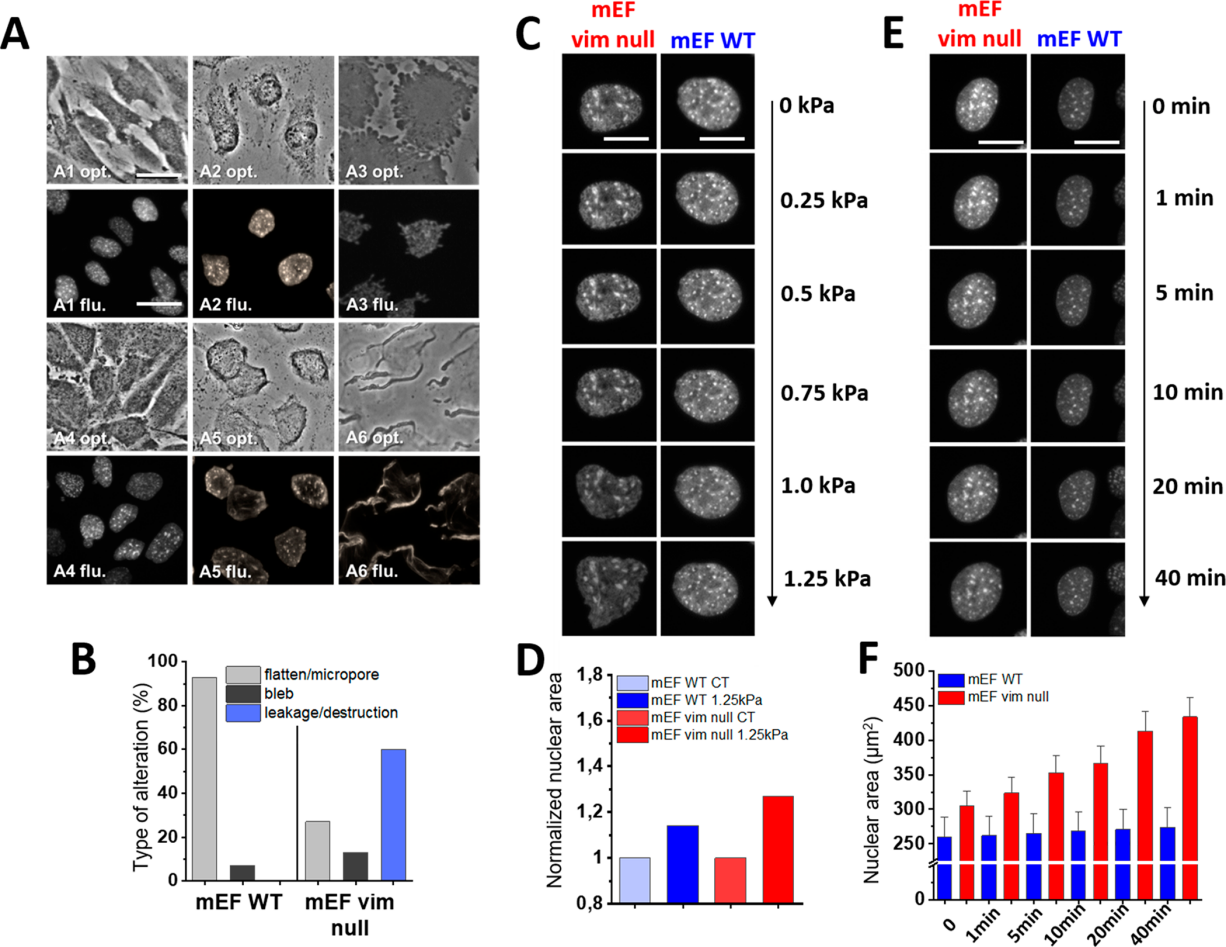
Compression-mediated alterations of the nuclei of wild type mEF cells (blue) and vim null mEF cells (red). (A) Representative images of different types of nuclei alterations in vim null mEF cells upon 1.25 kPa compression: A1, control sample; A2, A3, nucleus flattening/micropores; A4, bleb formation; A5, A6, leakage/nuclei membrane destruction (opt., optical image; flu., fluorescence image; scale bar, 30 μm). (B) The percentage of dominant nuclear alterations for wild type mEF and vim null mEF after compression. (C) Compression-mediated alterations of wild type and vim null mEF cells as the function of increasing compressive force (scale bar 20 μm). (D) Normalized nuclear area for compressed wild type and vim null mEF cells. (E) Compression-mediated alteration of wild type and vim null mEFs at a constant compressive force of 0.25 kPa and increasing time of compression (scale bar 20 μm). (F) The changes in nuclear area at the constant compressive force of 0.25 kPa and increasing time of compression.

Figure 4A shows representative images of different types of nuclear deformation and damage to the nuclear membrane in vim null mEFs, ranging from the rounded shape of an uncompressed nucleus (A1), an intact but flattened nucleus (A2, A3), blebbing nucleus (A4) to nuclear membrane disruption and chromatin leakage (A5, A6) that are quantified on Figure 2B. Figure 4C,D shows that the nuclei of vimentin null fibroblasts become significantly more compressed than wild type ones over a range of stresses from 0.25 to 1.25 kPa, which are relevant to *in vivo* pressure gradients in tissues. Figure 4E,F provides a quantitative estimate of the degree of nuclear damage in response to a constant compressive load of 0.25 kPa when applied over time. In both cases (Figure 4C,E), nuclei in wild type mEFs undergo very little damage with less than 5% of nuclei showing any evidence of blebs and none with disrupted nuclear membranes. In contrast, vimentin null fibroblasts have almost twice as many nuclei with blebs, and more than 30% of the nuclei show disruption of the nuclear membrane.

Intermediate filaments acquired their name because they were intermediate in size between the thin filaments (F-actin) and thick filaments (acto-myosin) of muscle cells.^21^ However, in almost all other respects they are largely outliers in terms of their chemistry and polymerization mechanisms and in their physical properties.^15^ Vimentin intermediate filaments have significantly more surface charge than F-actin of microtubules or even other types of intermediate filament such as keratins.^14^ In addition, their unusual structure, characterized by loose alignment of many alpha helical coiled coils, produces filaments that are much softer to bending than to stretching. In contrast, the stiffness of F-actin and microtubules is well described by a single stiffness parameter when they are deformed in either bending or stretching.^22^ The unique physical properties of intermediate filament networks have been noted previously; they have shear moduli much smaller than those of F-actin filaments when measured at small strains, but they become much more stiff when deformed to large shear strains and resist breakage,^23,24^ which is a critical feature of the much more brittle F-actin and microtubules networks.^25^ This combination of increased flexibility and large surface charge leads to two important features of VIF networks. The flexibility means that filament buckling, which accounts for the softening of more rigid fiber networks,^6^ does not occur, and therefore this mechanism for compression softening is not available. In addition, the large surface charge prevents long-term changes in volume, and therefore as seen in Figure 1D uniaxial deformations of these networks in either compression or extension leads to increased stretching of filaments, which is a mechanism that to leads to the increased shear modulus. Vimentin was also reported to impact relaxation of *in vitro* multicomponent interpenetrating cytoskeletal networks^26^ and increase cell elastic behavior for large and repeated deformations.^16^ Vimentin networks are not passive elements of the cell cytoskeleton and their dynamics is regulated by multiple signaling pathways, in response to cell mechanical stress and when the cell performs its physiological functions, like proliferation, migration, or apoptosis.^27^ Many intermediate filament-associated proteins can act as molecular motors or vimentin cross-linkers, such as adenomatous polyposis coli (APC) required for microtubule dependent rearrangements of vimentin network during migration or kinesin-1, dynein, and plectin that participate in vimentin assembly.^28−30^

The unique contribution of vimentin networks, their stiffening in compression, and their concentration around the nucleus were previously shown to protect the nucleus against damage as cells squeeze through narrow spaces.^20^ The importance of protecting the nucleus against compressive stress might also be related to the fact that intermediate filaments are not expressed in plant or fungal cells, where compressive stresses are taken up by the rigid cell wall that provide nucleus protection. Although our experiments were performed on single, isolated cells residing in an environment that is far from physiological, the effects explored using AFM-based whole cell compressions show the uniqueness of vimentin networks in preserving nucleus mechanical integrity and cell resilience upon large compressive strains.

## Materials and Methods

### Protein Purification

Actin was purified from rabbit skeletal muscle,^31^ and biotin-modified actin was purchased from Molecular Probes (Eugene, OR, U.S.A.). Monomeric actin was prepared by dialysis against 5 mM Tris-HCl pH 8.0 and 0.2 mM CaCl_2_. Actin was polymerized by addition of 2 mM Mg^2+^ and 100 mM KCl and cross-linked by addition of avidin (Sigma-Aldrich, St. Louis MO, U.S.A.) as previously described.^32^ Vimentin purified from Ehrlich ascites tumor cells was provided by Peter Traub^33^ polymerized at 4 mg/mL as previously described^25^ and cross-linked with 2 mM Cu^2+^, which has been shown to be an efficient cross-linker of vimentin filaments and other similarly charged polyelectrolyte filaments.^34^ Microtubule protein (MTP) was purified from bovine brain through cycles of temperature-induced polymerization and depolymerization as previously described and pellet by centrifugation.^35,36^ Further details can be found in Supporting Information.

### Rheological Measurements of the Cytoskeletal Networks under Uniaxial Compression

Measurements of the dynamic shear storage modulus (*G*′) at 2% shear strain and 1 Hz frequency were made with an RFS3 (TA Instruments, New Castle, DE, U.S.A.) or a Kinexus (Malvern Instruments, Malvern, U.K.) rheometer equipped with 20 mm diameter parallel plates. Further details can be found in Supporting Information.

### Modeling of the Cytoskeletal Networks under Uniaxial Compression

To explore whether incompressibility can explain the stiffening observed in the experiments, we calculate the expected dependence of the shear modulus on axial strain for a gel of semiflexible filaments^37^ in the relevant rheometer geometry, with and without imposing sample incompressibility. Specifically, we consider a cylindrical gel sample of initial height *h* and radius *R* in a parallel plate rheometer, to which uniaxial strain *ε*_*z*_ is first applied by varying the distance between the plates, after which shear strain γ is applied by twisting the upper plate while the lower plate remains fixed. We consider two possibilities: (1) the gel is entirely compressible and undergoes homogeneous uniaxial strain, or (2) the gel is incompressible and bonded to the upper and lower plates, such that the compressed sample bulges outward at the edges with a parabolic profile.^38−40^ Sketches of the two deformation varieties are shown in Figure 1D. Further details on calculations can be found in Supporting Information.

### Cell Culture

Two cell lines were used, wild-type mouse embryonic fibroblasts (mEF WT) and vimentin-null fibroblast (mEF vim null). Cells were cultured in DMEM (ATCC) with 10% fetal bovine serum (FBS), penicillin (50 μg/mL) and streptomycin (50 μg/mL). Cells were maintained at 37 °C in an atmosphere containing 5% CO_2_ with saturated humidity.

### Whole Cell Compression Experiments

Experiments with whole cell compression of wild-type mouse embryonic fibroblasts (mEF WT) and vimentin-null fibroblasts (mEF vim null) were performed using a JPK Nanowizard 4 atomic force microscope equipped with cantilevers of nominal stiffness 2.4 N m^–1^ with a 25 μm diameter sphere attached (Novascan) according to previously published protocols.^9,41^ Both round and spread cells were measured with 20 cells per condition tested. Further details can be found in Supporting Information.

### Nucleus Isolation

Nuclei were isolated as previously described^42,43^ with the modifications detailed in Supporting Information.

### AFM Measurements of Isolated Nuclei and Cytoplasts

AFM measurements were made with a DAFM-LN Bioscope (Veeco, Woodbury, NY) mounted on an Axiovert 100 microscope (Zeiss, Thornwood, NY). Isolated nuclei were compressed using tipless silicon nitride cantilevers (NP-O10, Bruker, Camarillo, CA) with a nominal spring constant of 0.12 N/m at 1 Hz and a maximum applied force of 16 nN. Cytoplasts were indented using silicon nitride cantilevers with a 1 μm diameter spherical bead attached and a spring constant of 0.06 N/m (Novascan, Boone, IA, U.S.A.). Indentations were made at three points in the center of the cytoplast at a frequency of 1 Hz and a maximum applied force of 6 nN. All measurements were made at room temperature in PBS (plus Ca and Mg) with a minimum of 20 cells per condition measured.

### Loading Test

The device used to assess alteration of cell nuclei under pressure consisted of two slides and a microscope holder (Supplementary Figure 1). Before measurements the top slide with cells was drained by gently touching the edge of the slide to a dust-free wipe to pull off excess medium. Selected pressures were converted into a unit of force. Weights in the shape of an upper slide were applied on top. For nuclei observation, a Zeiss Axio Observer D1 microscope was used. Further details of the loading tests performed are found in Supporting Information.

### Statistical Analysis

The significance of differences was determined using the two-tailed Student’s *t* test. Statistical analyses were performed using OriginPro 9.65 (OriginLab Corporation, Northampton, MA, U.S.A.). *p* < 0.05 was considered to be statistically significant. Data are presented as mean values ± SD.

## Supporting Information Available

Video of fluorescent vimentin cage undergoing deformation when the AFM tip compresses the cell (AVI)
Materials and methods, all necessary definitions, and Supplementary Figure 1 (PDF)

## Supplementary Material

nl2c00736_si_001.avi
