# Supplementary material for: Unique Role of Vimentin Networks in Compression Stiffening of Cells and Protection of Nuclei from Compressive Stress
Source: Nano Lett. 2022 Jun 9;22(12):4725–32. doi: 10.1021/acs.nanolett.2c00736 (PMC9228066; doi:10.1021/acs.nanolett.2c00736)
Supplement: Supplementary file 2 — nl2c00736_si_002.pdf [file nl2c00736_si_002.pdf]

Supporting information

Unique role of vimentin networks in compression stiffening of cells and protection of nuclei from compressive stress

Katarzyna Pogoda[1*], Fitzroy Byfield[2], Piotr Deptuła[3], Mateusz Cieśluk[3], Łukasz Suprewicz[3], Karol Skłodowski[3], Jordan L. Shivers[4,5], Anne van Oosten[2], Katrina Cruz[2], Ekaterina Tarasovetc[6], Ekaterina L. Grishchuk[6], Fred C. MacKintosh[4,5,7], Robert Bucki[3], Alison E. Patteson[8], Paul A. Janmey[2*]

1. Institute of Nuclear Physics Polish Academy of Sciences, Krakow, PL-31-342, Poland
2. Department of Physiology, and Institute for Medicine and Engineering, University of Pennsylvania, Philadelphia, PA 19104-6383, USA
3. Department of Medical Microbiology and Nanobiomedical Engineering, Medical University of Bialystok, PL-15222 Bialystok, Poland
4. Department of Chemical and Biomolecular Engineering, Rice University, Houston, TX 77005, USA
5. Center for Theoretical Biological Physics, Rice University, Houston, TX 77030, USA
6. Department of Physiology, University of Pennsylvania, Philadelphia, PA 19104-6383, USA
7. Departments of Chemistry and Physics & Astronomy, Rice University, Houston, TX 77005, USA
8. Department of Physics and BioInspired Institute, Syracuse University, Syracuse, NY 13210, USA

*Corresponding author(s):

Katarzyna Pogoda: katarzyna.pogoda@ifj.edu.pl

Paul A. Janmey: janmey@pennmedicine.upenn.edu

Definitions

Given the displacement functions for the gel corresponding to the application of strain by the rheometer, which transform the initial material coordinates (r', θ', z') to the deformed coordinates (r, θ, z),

$$z = z(r', \theta', z')$$
$$r = r(r', \theta', z')$$
$$\theta = \theta(r', \theta', z')$$

we can compute the deformation gradient tensor [1]

$$\boldsymbol{F} = \begin{pmatrix} \partial r/\partial r' & (1/r')\partial r/\partial \theta' & \partial r/\partial z' \\ r\partial \theta/\partial r' & (r/r')\partial \theta/\partial \theta' & r\partial \theta/\partial z' \\ \partial z/\partial r' & (1/r')\partial z/\partial \theta' & \partial z/\partial z' \end{pmatrix}$$

For some set of boundary conditions and applied deformation gradient tensor $\boldsymbol{F}$, the mechanical response of the gel, as measured by the rheometer, can be determined from the total stress $\boldsymbol{T} = -p\boldsymbol{I} + \boldsymbol{\sigma}$, in which the hydrostatic pressure p and stress tensor $\boldsymbol{\sigma}$ are, in general, functions of r and z (assuming rotational symmetry). The relevant components of $\boldsymbol{T}$ are

$$T_{rr} = -p + \sigma_{rr}$$
$$T_{\theta\theta} = -p + \sigma_{\theta\theta}$$
$$T_{zz} = -p + \sigma_{zz}$$
$$T_{rz} = \sigma_{rz}$$
$$T_{\theta z} = \sigma_{\theta z}$$

Assuming that the gel is in static equilibrium and that gravity can be neglected, we have $\nabla \cdot \boldsymbol{T} = 0$, or in cylindrical coordinates with $\partial(\,)/\partial\theta = 0$,

$$\frac{\partial}{\partial r}(rT_{rr}) + r\frac{\partial}{\partial z}(T_{rz}) - T_{\theta\theta} = 0$$
$$\frac{\partial}{\partial r}(rT_{r\theta}) + r\frac{\partial}{\partial z}(T_{\theta z}) = 0$$
$$\frac{\partial}{\partial r}(rT_{rz}) + r\frac{\partial}{\partial z}(T_{zz}) = 0$$

Compressible gel with zero transverse strain

We first consider the mechanical response of a fully compressible gel subject to homogeneous uniaxial strain ε_z followed by torsion by angle θ. In cylindrical coordinates, the displacement functions describing this deformation are

$$z = (1 + \varepsilon_z)z'$$
$$r = r'$$
$$\theta = \theta' + \gamma r'z = \theta' + \gamma r'(1 + \varepsilon_z)z'$$

in which $\gamma = \theta/h$ [1]. Since $\boldsymbol{F} = \boldsymbol{F}(r)$ in this case, we can compute the apparent shear modulus as

$$G(\varepsilon_z) = \lim_{\gamma \to 0^+} \frac{4}{\gamma R^4} \int_0^R r^2 \sigma_{\theta z}(r, \varepsilon_z, \gamma) dr.$$

Bonded incompressible gel

Now, we assume that the gel is incompressible and rigidly bonded to the upper and lower plates of the rheometer, so that under compression it bulges outward with a parabolic profile [2]–[4]. At the midplane, enforcing volume preservation moves a point at radius r' to

$$r_{\max}(\varepsilon_z) = \frac{1}{4}\left(\sqrt{\frac{5(5-\varepsilon_z)}{1+\varepsilon_z}} - 1\right) r'$$

with $r_{\max} \approx (1 - 3\varepsilon_z/4) r'$ for small strains [2]. The displacement functions are

$$\begin{aligned}
z &= \lambda_z(z')z' \\
r &= \left(1 + 5\left(\sqrt{\frac{1-\varepsilon_z/5}{1+\varepsilon_z}} - 1\right)\left(\left(1 - \frac{z}{h(1+\varepsilon_z)}\right)\frac{z}{h(1+\varepsilon_z)}\right)\right) r' \\
&= \left(1 + 5\left(\sqrt{\frac{1-\varepsilon_z/5}{1+\varepsilon_z}} - 1\right)\left(\left(1 - \frac{\lambda_z(z')z'}{h(1+\varepsilon_z)}\right)\frac{\lambda_z(z')z'}{h(1+\varepsilon_z)}\right)\right) r' \\
\theta &= \theta' + \gamma r' z = \theta' + \gamma r' \lambda_z(z') z'
\end{aligned}$$

in which $\lambda_z = 1 + (2 - 3(z'/h))(z'/h)\varepsilon_z$ [3]. The deformation gradient tensor in this case varies as a function of both r and z. The apparent shear modulus is computed as

$$G(\varepsilon_z) = \lim_{\gamma \to 0^+} \frac{4}{\gamma R^4 h} \int_0^h \int_0^R r^2 \sigma_{\theta z}(r, z, \varepsilon_z, \gamma) dr dz$$

For a given compression or extension applied to the bonded gel, the largest principal strain occurs adjacent to the plates $z = [0, h]$ at the outer edge, where shear is maximal and depends on the gel aspect ratio R/h.

Bibliography for ***Definitions*** *section*

[1] C. W. Macosko and R. G. Larson, *Rheology: Principles, Measurements, and Applications*. Wiley, 1994.
[2] A. N. Gent and P. B. Lindley, "The compression of bonded rubber blocks," *Proc. Inst. Mech. Eng.*, vol. 173, no. 3, pp. 111–122, 1959.
[3] S. Qiao and N. Lu, "Analytical solutions for bonded elastically compressible layers," *Int. J. Solids Struct.*, vol. 58, pp. 353–365, 2015.
[4] M. T. J. J. M. Punter, B. E. Vos, B. M. Mulder, and G. H. Koenderink, "Poroelasticity of (bio)polymer networks during compression: theory and experiment," *Soft Matter*, vol. 16, no. 5, pp. 1298–1305, 2020.

Materials and methods

Protein purification

Microtubule protein (MTP) purification. Fresh bovine brains were cleared from meninges and blood vessels and homogenized using blender in ice-cold assembly buffer (0.1 M 2-(N-morpholino)ethanesulfonic acid, 1 mM EGTA, 1 mM $MgSO_4$, 1 mM Mg-ATP, pH 6.4). The homogenate was centrifuged at 2,340 x g for 15 min at 4°C. The supernatant was collected and supplemented with 25% ice-cold glycerol, followed by ultracentrifugation in 50.2 Ti Beckman rotor at 165,000 x g for 35 min at 4°C to pellet residual insoluble parts. The supernatant was collected and supplemented with 2 mM Mg-GTP. Next, the microtubules were polymerized for 40 min at 37°C and pelleted by centrifugation at 165,000 x g for 35 min at 37°C. Microtubule pellets were resuspended in ice-cold assembly buffer, homogenized using Dounce homogenizer and depolymerized on ice for 30 min. The solubilized proteins were supplemented with 25% glycerol and 2 mM GTP and clarified by centrifugation at 165,000 x g for 35 min at 4°C. The supernatant was collected to obtain MTP (4-8 mg/ml), containing tubulin with assembly-promoting/stabilizing microtubule-associated proteins, such as MAP2 and tau (1). Prior to each experiment, an aliquot with MTP was polymerized at 37°C for ~ 30-60 min (2).

Modelling of the cytoskeletal networks under uniaxial compression

Assuming that the deformation is affine, the local strain is determined by the deformation gradient tensor $\boldsymbol{F} = \boldsymbol{F}(r, z, \varepsilon_z, \gamma)$, defined as $F_{ij} = \partial x_i / \partial X_j$, which transforms points in the gel from the initial coordinates $\boldsymbol{X} = (r', \theta', z')$ to the deformed coordinates $\boldsymbol{x} = (r, \theta, z) = \boldsymbol{x}(\boldsymbol{X})$. The total stress at each point is $\boldsymbol{T} = -p\boldsymbol{I} + \boldsymbol{\sigma}$, in which the hydrostatic pressure p and network stress tensor

$\boldsymbol{\sigma} = \boldsymbol{\sigma}(\boldsymbol{F})$ are, in general, functions of r and z, assuming rotational symmetry. For a configuration with length density (filament length per unit of volume) ρ, the network stress tensor is

$$\sigma_{ij} = \rho \left\langle \tau(\ell) \frac{F_{il} n_l F_{jk} n_k}{|\boldsymbol{F}\hat{\boldsymbol{n}}|} \right\rangle \tag{1}$$

in which the average is taken over all initial filament orientations $\hat{\boldsymbol{n}}$. We assume that the filaments are isotropically oriented in the undeformed configuration. For semiflexible filaments with persistence length ℓ_p, contour length ℓ_c, and enthalpic spring constant μ, the average end-to-end distance ℓ can be expressed as a function of the applied tension τ as (3), (4),

$$\ell(\tau) = (1 + \tau/\mu)\ell_c - \frac{k_b T}{2\tau} \left(\frac{\ell_c \sqrt{(1 + \tau/\mu)\tau/(k_B T \ell_p)}}{\tanh\left(\ell_c \sqrt{(1 + \tau/\mu)\tau/(k_B T \ell_p)}\right)} - 1 \right). \tag{2}$$

We assume that the initial end-to-end distance of the filament satisfies $\tau = 0$, for which $\ell_0 = \ell(0) = \ell_c - \ell_c^2/(6\ell_p)$ (3), so that the initial configuration of the gel is unstressed. We can thus write the strained end-to-end distance as $\ell = \ell_0 |\boldsymbol{F}\hat{\boldsymbol{n}}|$. For finite strains, we compute $\tau(\ell)$ by numerical inversion of Eq. 2. Expanding τ to linear order in the filament extension $\lambda = (\ell - \ell_0)/\ell_0$ about $\lambda = 0$, we have $\tau \sim k\lambda$, with $k = \left((1/\ell_0)(\ell_c^4/(90 k_B T \ell_p^2)) + 1/\mu\right)^{-1}$. An isotropic material composed of springs with length density ρ and spring constant k (such that the force extension relation is $\tau = k\lambda$) has shear modulus $G_0 = \rho k/15$. Therefore, specifying ℓ_c, ℓ_p, μ, T, and G_0 sets the length density $\rho = 15 G_0 \left((1/(\ell_c - \ell_c^2/(6\ell_p)))(\ell_c^4/(90 k_B T \ell_p^2)) + 1/\mu\right)$. From the torque $M(\varepsilon_z, \gamma)$ imparted on the upper plate by the gel,

$$M(\varepsilon_z, \gamma) = 2\pi \int_0^R T_{\theta z}(r, z' = h, \varepsilon_z, \gamma) dr\,, \tag{3}$$

we compute the apparent shear modulus

$$G(\varepsilon_z) = \lim_{\gamma \to 0} \frac{2M(\varepsilon_z, \gamma)}{\pi R^4 \gamma}\ . \tag{4}$$

We assume a sample radius $R = 10$ mm, initial plate-to-plate distance $h = 3$ mm and temperature $T = 298$ K, with the following parameters depending on the type of biopolymer:

	ℓ_c (μm)	ℓ_p (μm)	μ (pN)	G_0 (Pa)
vimentin (Fig. 1B)	2	0.5	0.15	25
hyaluronic acid (Fig. 1C)	0.023	0.005	500	300

In Fig. 1B-C, we plot the apparent shear modulus G as a function of applied uniaxial strain ε_z for the parameters specified above, along with the experimental data for both vimentin and hyaluronic acid. Whereas the fully compressible model softens (exhibiting decreasing G) under compression, enforcing local volume conservation gives rise to compression-driven stiffening (increasing G) comparable with the experimental data. In the model, this stiffening occurs because the incompressibility condition leads to stretching of certain regions of the sample, despite the macroscopic applied compression, which increases the stiffness of the constituent semiflexible filaments. Note that the maximum extension corresponds to the largest of the three principal strains, λ_i, which are determined by the eigenvalues λ_i^2 of the right Cauchy-Green tensor $\boldsymbol{C} = \boldsymbol{F}^T\boldsymbol{F}$.

Rheological measurements of the cytoskeletal networks under uniaxial compression

The protein or polysaccharide solutions were added between rheometer plates prior to polymerization. After polymerization, the appropriate buffer was added around the free edge of the sample to prevent drying and allow fluid to flow in or out of the sample. Static axial strain was imposed by changing the gap between the plates as previously described (5, 6) . Compression of the sample led to an immediate axial stress, which decayed with time and was monitored until it reached a steady value (typically 2-5 minutes), at which time the dynamic shear storage modulus was measured. All measurements were done at room temperature except for microtubules, which were measured at 37 ^{0}C.

Whole cell compression experiments

For round cell experiments, cells were trypsinized in order to round up and detach from the surface of the TC flask. Next, cells were centrifuged, resuspended in growth medium, and round cells were immediately placed on a Petri dish that was mounted on the AFM stage and indented uniaxially with a constant force of 450 nN at a speed of 5 μm s^{-1} as follows: (i) the AFM cantilever was placed over the rounded cell as controlled visually through the optical microscope, (ii) the point of contact between the cantilever and cell surface was recorded and assumed to be the cell height, (iii) each cell was indented until 450 nN force was reached and data were saved automatically as force (nN) *vs.* distance curves (μm). For spread cells, the same procedure was performed using mEF WT and mEF vim null cells that were seeded 24h prior to experiments and were fully spread. The force (nN) *vs.* distance curves (μm) were then converted into stress (kPa) *vs.* cell height (%) with the assumption that normal stress can be calculated as the ratio of the applied force (F) to the area of deformation. The area of deformation A was calculated as a spherical cap, or $A = 2\pi rh$ where r is the radius of the sphere and h is the depth at which cell was indented. The cell height percentage was calculated as the percentage of the total cell height that underwent indentation at a given force. Assuming that the strain is 0% at 100% cell height, then the cell height can be converted to a strain percentage by subtracting the cell height percentage from 100%. Finally, the stress is then given by the ratio of the force to the area of deformation. The data were obtained from 10 cells and averaged, with the error bar denoting the standard deviation. The same measurement allowed us to determine cell height. The above protocol was repeated using NIH 3t3 fibroblasts expressing GFP-vimentin together with time-lapse video of the fluorescent vimentin cage that undergoes deformation when the AFM tip compresses the cell (Fig. 2G, Supplementary video 1).

Nucleus isolation

18 mm glass coverslips were attached to PDMS rings (inner diameter:13mm, outer diameter:20 mm, height :3mm) to make inserts for thick-walled polycarbonate centrifuge tubes (32 ml capacity, Beckman Coulter, Brea, CA). To stabilize inserts during centrifugation, PDMS was cured within centrifuge tubes to form a flat surface perpendicular to the centrifuge tube wall (Figure 3A). Inserts were cleaned by rinsing w/ dd H20 and ethanol then exposed to air plasma for 3 minutes, sterilized under UV for 20 mins then coated overnight with 0.1 mg/ml rat tail collagen diluted in PBS. Wild type and vimentin null MEFs were then cultured on the inserts by adding 200 ul of cell suspensions

at a concentration of 2 x 10^^{5} cells/ml, per insert overnight. Immediately before enucleation, cell nuclei were stained with 5 ug/ml Hoechst 33342 (Molecular probes, Eugene, OR) for 30 mins at 37 degrees. Cells on inserts were then rinsed w/PBS and placed face down in centrifuge tubes containing 2 ug/ml cytochalasin D (Sigma Aldrich, St. Louis, MO) dissolved in DMEM (plus 1% FBS, 25 mM HEPES) and centrifuged at various speeds up to 2880 x g for up to 50 mins at 37 ^{o}C in an Optima LE-80K ultracentrifuge using an SW28 rotor (Beckman Coulter, Brea, CA). Inserts coated with 0.1 mg/ml poly-d-lysine were positioned under inserts with cells during centrifugation to collect released nuclei. Nucleus isolation efficiency was determined by imaging Hoechst staining of cells after centrifugation. Isolated nuclei were stored in cell culture media (DMEM plus 10% FBS, 25 mM HEPES) at 4 degrees while cells without nuclei (cytoplasts) were kept at 37 ^{o}C, also in cell culture media.

Loading test

Detailed information on the setup used for loading tests can be found on *Supplementary Figure 1*. For these experiments, 1x10^4 wild-type mouse embryonic fibroblasts (mEF WT) and vimentin-null fibroblast (mEF vim null) were seeded onto glass coverslip and maintained for 4 days until full confluence was reached. Then, cells were stained with 4′,6-diamidino-2-phenylindole (DAPI) to visualize cell nuclei. After vital staining, the supernatant was removed, and cells washed twice with PBS. Samples were measured immediately after staining. In the first experiment each control plate with mEF WT and mEF vim null was pressed using 1.25 kPa for 10 second and then the alteration of nuclei was observed. In the second experiment each control plate with mEF WT and mEF vim null was pressed in five steps using loads from 0.25 to 1.25 kPa. Photos after each of the five 5-second loads were taken. A confocal microscope was used to measure the distance between the slides after each load stage, which precisely determined the distance between the slides' surfaces. ImageJ was used for the quantitative analysis of nuclei.

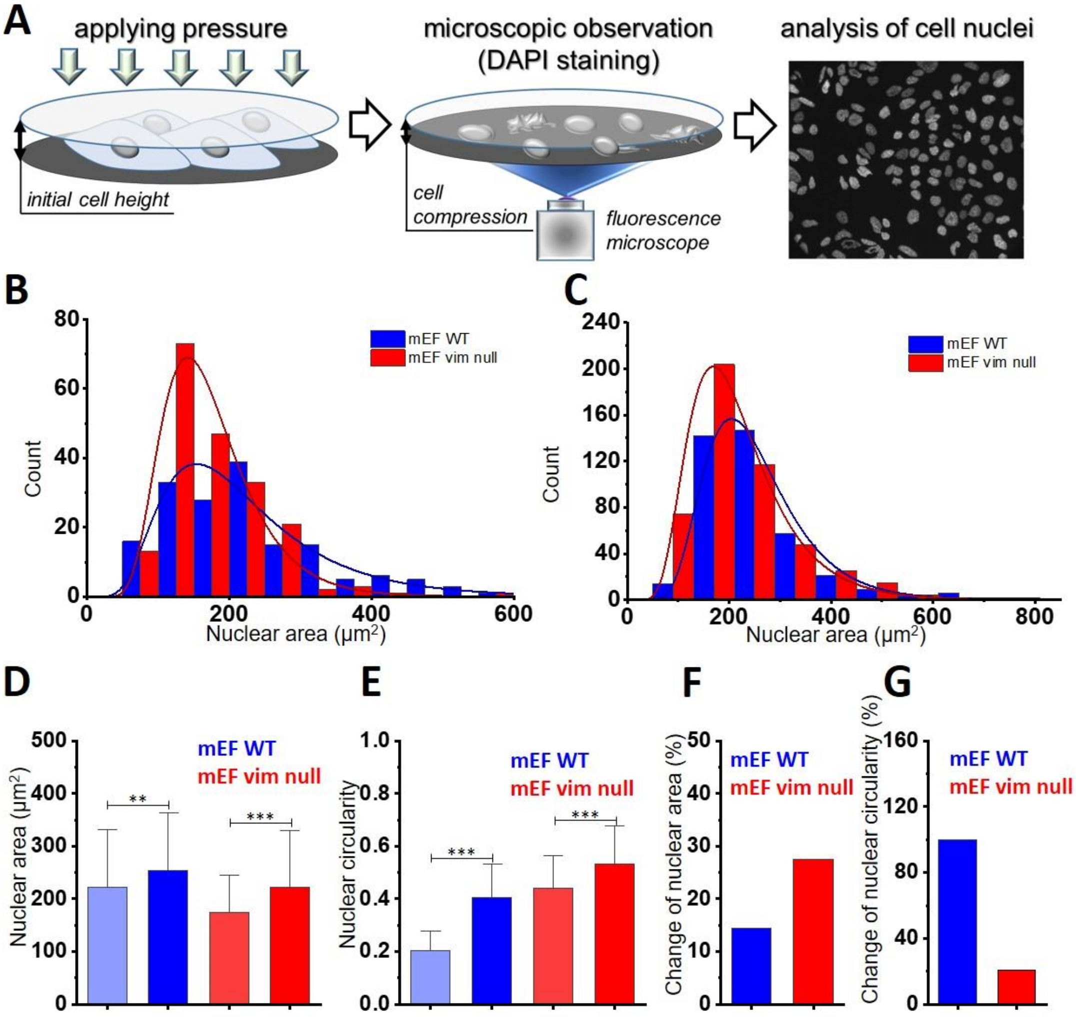

Supplementary Figure 1. Pressure-mediated alterations of two types of nucleus, isolated from mEF WT and mEF vim null. Panel A shows schematic representation of experimental setting that was used to assess alterations of the cell nuclei. Cell subjected to pressure were stained with DAPI to visualize nuclei morphology. Percentage of altered nuclei and types of alteration were determined based on 200 nuclei analyzed for each cell line. Panel B shows the nuclear area values distributions obtained for control (uncompressed) cells. Panel C shows the nuclear area values distributions obtained for cells after stress was applied (1.25 kPa). Probability density function of the log normal distribution was fitted. The blue column represents mEF WT cells and the red column represents mEF vim null cells. Panels D and E show mean values of nuclear area and nuclear circularity for control and compressed (1.25 kPa) WT mEF and vimentin null mEF. Panel F corresponds to the nuclear area distributions and indicates percentage increase of nuclear area

upon applied compressive stress. A greater increase of nuclear area for mEF vim null was observed. Panel G shows percentage increase of nuclear circularity upon applied stress. A greater increase of nuclear circularity for WT mEF was observed.

Bibliography for ***Materials and Methods*** *section*

1. H. P. Miller, L. Wilson, Preparation of microtubule protein and purified tubulin from bovine brain by cycles of assembly and disassembly and phosphocellulose chromatography. *Methods in cell biology* **95**, 3-15 (2010).
2. M. Chakraborty, E. V. Tarasovetc, E. L. Grishchuk, In vitro reconstitution of lateral to end-on conversion of kinetochore-microtubule attachments. *Methods Cell Biol* **144**, 307-327 (2018).
3. C. Storm, J. J. Pastore, F. C. MacKintosh, T. C. Lubensky, P. A. Janmey, Nonlinear elasticity in biological gels. *Nature* **435**, 191-194 (2005).
4. C. P. Broedersz, F. C. MacKintosh, Modeling semiflexible polymer networks. *Rev Mod Phys* **86**, 995-1036 (2014).
5. A. S. van Oosten *et al.*, Uncoupling shear and uniaxial elastic moduli of semiflexible biopolymer networks: compression-softening and stretch-stiffening. *Sci Rep* **6**, 19270 (2016).
6. A. S. G. van Oosten *et al.*, Emergence of tissue-like mechanics from fibrous networks confined by close-packed cells. *Nature* **573**, 96-101 (2019).
